# Substituting Whole Wheat Flour with Pigeon Pea (*Cajanus cajan*) Flour in Chapati: Effect on Nutritional Characteristics, Color Profiles, and In Vitro Starch and Protein Digestion

**DOI:** 10.3390/foods11203157

**Published:** 2022-10-11

**Authors:** Sirin Sachanarula, Praew Chantarasinlapin, Sirichai Adisakwattana

**Affiliations:** Phytochemical and Functional Food Research Unit for Clinical Nutrition, Department of Nutrition and Dietetics, Faculty of Allied Health Sciences, Chulalongkorn University, Bangkok 10330, Thailand

**Keywords:** pigeon pea, whole wheat flour, starch and protein digestibility, plant-based alternative

## Abstract

Pigeon pea (*Cajanus cajan* (L.) Millsp.), a potential legume as an economical source of protein, is commonly cultivated in tropical and subtropical regions of the world. Therefore, pigeon pea may be potentially used as a substitute to improve the nutritional profile of foods. In the present study, the effect of substitution of whole wheat flour (WWF) with 20% and 40% pigeon pea flour (PPF) on the nutritional properties, color profiles, and starch and protein digestibility of chapati was investigated. The results showed that PPF had higher protein content but less carbohydrate than WWF. The protein content of chapati substituted with 20% and 40% PPF increased by 1.18 and 1.34 times, respectively, compared to WWF chapati, along with a marked decrease in carbohydrate content. Analyses further revealed an increase in the lightness and yellowness and a decrease in the redness of the chapati. Furthermore, glucose release from chapati with 20% and 40% PPF under simulated digestion was attenuated, corresponding to decreased hydrolysis and a predicted glycemic index. In the 40% PPF chapati, a significant reduction in slowly digestible starch (SDS) with increased resistant starch (RS) proportions was achieved without altering the effect on rapidly digestible starch (RDS). In addition, the level of amino-group residues was markedly elevated in 20% and 40% PPF substituted chapati compared to WWF chapati. These findings suggest that PPF can serve as a promising plant-based alternative ingredient to improve the nutrient value of chapati by reducing starch and increasing protein digestibility.

## 1. Introduction

Plant-based alternatives have been increasingly recommended to minimize the consumption of animal-based foods [1]. Several studies have shown the beneficial effects of incorporating plant-based diets into clinical management, such as type 2 diabetes [2], dyslipidemia [3], and weight control [4]. Interestingly, the consumption of plant-based diets has also been associated with a decreased risk of incident cardiovascular disease and mortality in middle-aged adults [5]. Generally, plant protein sources contribute up to 65% of the world’s edible protein supply [6], such as cereal grains, legumes, vegetables, fruits, and nuts, with cereal grains accounting for 47% [7]. Wheat is the most widely cultivated cereal that has become the world’s leading plant-based food ingredient and the economic backbone in many countries. Chapati, an unleavened flatbread produced by grinding wheat without separating the germ and bran from the endosperm (whole wheat), is a popular staple food in South Asian nations [8]. Owing to the increased awareness and modern concept of a healthier diet, the substitution of whole wheat flour with other natural ingredients to enhance the nutritional status of chapati has become more popular. For example, substituting wheat with defatted rice bran improved the nutritional characteristics by increasing the crude protein, fat, and dietary fiber content [9]. Existing data show that chapatis made from composite blends (wheat and finger millet flour) had higher levels of soluble starch and soluble amylose, as well as slower digestible starch (SDS) and resistant starch (RS) [10]. A recent study demonstrated that consumption of vegetable powder and bean powder–supplemented chapatis reduced postprandial glucose and insulin in healthy participants [11]. Thus, growing interest has been dedicated to identifying alternative plants that can be used as whole wheat flour substitutes in making chapati to provide physiological relevance and health benefits.

Pigeon pea, *Cajanus cajan* (L.) Millsp. is a legume crop grown widely in Africa, Central America, and India [12]. It has been commonly consumed in the human diet because this plant has the highest percentage of protein, up to 24%, and is especially a rich source of lysine [13]. Various reports have shown that pigeon pea can be used as a novel ingredient in restructured meat products [14,15]. It is also used as a substitute for wheat flour to improve the nutritional value of biscuits [16] and crackers [17]. The composite biscuits made by blending pigeon pea and wheat flour had a low glycemic index, which causes a reduced peak in postprandial glucose in diabetic patients after 30 min of consumption [18]. Although pigeon pea, as a potential food ingredient in wheat flour, has been previously studied, the effect of pigeon pea flour substitution on the starch and protein digestibility of whole wheat flour is limited. This gap in the literature has motivated the present study to determine the effect of substituting whole wheat flour (WWF) with pigeon pea flour (PPF) on the nutritional characteristics, color profiles, and digestion of starch and protein in chapati. We hypothesized that PPF substitution in whole wheat flour–based chapati could enhance nutrient and color profiles, reduce carbohydrate digestion, and improve protein content.

## 2. Materials and Methods

### 2.1. Materials

Pigeon pea was obtained from the local market, Tak, Thailand. Pepsin from porcine gastric mucosa powder, α-amylase Type VI-B from porcine pancreas, and pancreatin from porcine pancreas were obtained from Sigma-Aldrich Chemical (St. Louis, MO, USA). Amyloglucosidase from *Aspergillus niger* was purchased from Roche Diagnostics (Indianapolis, IN, USA). Glucose LiquiColor^®^ was purchased from HUMAN GmbH (Wiesbaden, Germany).

### 2.2. Pigeon Pea Flour

Pigeon pea was made into flour following a previous method, with slight modifications [17]. Briefly, the dry seeds were cleaned, handpicked, boiled for 1 min, and then soaked in that water for 1 h and manually dehulled. The dehulled seeds were then blended in a grinder (DXM-500, DXFill Machine, Samut Prakan, Thailand) into a slurry paste, spread on a tray lined with aluminum foil, and dried in an air-drying oven at 65 °C for 14 h. After the drying process, the flour was blended in a tilted-head stand mixer (Model 5K45SS Heavy Duty, KitchenAid, Greenville, OH, USA) to acquire homogenous samples. The flour blends were then sieved through a 150 mm screen mesh and stored in an aluminum zip-lock bag at room temperature until use. 

### 2.3. Preparation of Chapati

Whole wheat flour (WWF) was replaced by pigeon pea flour (PPF) at 20% and 40%. First, a dough was formed by mixing flour (100 g) with 60 mL of room temperature water using an electric mixer (Model 5K45SS Heavy Duty, KitchenAid, Greenville, OH, USA) for 5 min until a dough was formed [18]. The final dough was hand-kneaded for 2 min, rested, and covered with a wet cloth for 30 min at room temperature before use. The dough was manually divided, weighed into 40 g pieces, and rolled out into a sheet 15 cm in diameter with a thickness of 2 mm. After that, the dough was heated using a non-stick pan preheated for 10 min on an electric stove set at max level (200 °C) (HW-116A2, House Worth, Bangkok, Thailand) for 30 s on each side. Finally, slight pressure was applied to the sheets until they puffed for 20 s; then, they were allowed to cool at room temperature [19]. 

### 2.4. Proximate Analysis

Protein, fat, total dietary fiber, moisture, and ash content in flour and chapati samples were determined by the Association of Official Analytical Chemists (AOAC) methods [20]. Total carbohydrate content and total calories were estimated by applying the following expressions [21]:

Total carbohydrate (%) = 100 − (%protein + %fat + %moisture + %ash)(1)

Total calories (kcal/100 g) = (%protein × 4) + (%total carbohydrate × 4) + (%fat × 9)(2)

### 2.5. Color Measurement

The color of chapati was measured using a colorimeter by the Hunter Lab Color Measuring System (Color–flex EZ, Hunter Lab, Reston, VA, USA). The instrument was calibrated using the standard tiles. Portions of flour blends and chapati were randomly selected and homogenized using a grinder (DXM-500, DXFill Machine, Samut Prakan, Thailand). Then, homogenous samples were placed in the sample holder, and the reflectivity was recorded in triplicate. The results were reported as an average and expressed according to the CIE L* a* b* system. The total color difference (∆*E*) between WWF and PPF samples was calculated using the following formula [22]:

∆*E* = [(∆L*)^2^ + (∆a*)^2^ + (∆b*)^2^]^1/2^ (3)

### 2.6. Starch Digestion

Starch digestion was performed according to the method outlined in a previous study, with slight modification [23]. Briefly, 500 mg of sample was mixed with 1 mL of artificial saliva containing porcine α-amylase (250 U/mL in 0.2 M carbonate buffer, pH 7) for 15–20 s, followed by 5 mL of pepsin (4500 U/mL at 1 mL/mL in 0.02 M HCl, pH 2), and incubated at 37 °C in a shaking water bath at 100 rpm for 1 h (gastric phase). The mixture was then neutralized by adding 5 mL of 0.02 M NaOH before adjusting the pH to 6 (25 mL of 0.2 M sodium acetate buffer). Following that, a 5 mL mixture of pancreatin (2 mg/mL in 0.2 M acetate buffer, pH 6) and amyloglucosidase (28 U/mL in 0.2 M acetate buffer) was added and incubated for 180 min (intestinal phase). Digesta were collected at the end of the gastric phase and at different time points in the intestinal phase (0–180 min). To halt enzymatic reactions, the digesta was immediately heated at 90 °C for 10 min before cooling to room temperature. The digesta was centrifuged at 10,000 rpm, 4 °C for 15 min in a benchtop centrifuge (ROTINA 380R, Hettich, Tuttlingen, Germany). The supernatant of the digesta was collected and kept at −20 °C until analysis.

The glucose content in the digesta was measured using an enzymatic colorimetric GOPOD method (Glucose LiquiColor^®^, HUMAN GmbH, Wiesbaden, Germany). In brief, the working reagent (500 μL) was mixed with the sample digesta (5 μL) and incubated at room temperature for 10 min. The absorbance was measured at 500 nm using a microplate reader (PowerWave XS2, BioTek, Winooski, VT, USA). Glucose (100 mg/dL) was used as a standard. The rate of starch digestibility was expressed as the glucose concentration at different time intervals (0, 10, 20, 30, 60, 90, 120, 150, and 180 min). The glucose values (0–180 min) were plotted as a line graph, and areas under curves (AUCs) were calculated using the trapezoidal rule. The hydrolysis index (HI) was calculated according to the following equation:

HI = (AUC_sample_/AUC_glucose_) × 100 (4)

The predicted glycemic indices (pGI) of the samples were estimated using the following equation [24,25]:

pGI = 39.71 + 0.549 HI(5)

### 2.7. Total Starch and Starch Fraction 

Total starch was determined based on the method previously reported by Goni et al., with a slight modification [26]. The sample (50 mg) was mixed with 6 mL of 2 M KOH and vigorously shaken for 30 min. Then, 3 mL of 0.4 M sodium acetate buffer pH 4.75 was added, and the pH was adjusted to 4.5 using 6 M HCl. Amyloglucosidase (3260 U/mL, 60 μL) was added to the mixture and incubated in a shaking water bath for 45 min at 60 °C. After that, 1 mL of the solution was collected and heated at 90 °C for 10 min to stop the enzyme reaction. The solution was cooled down to room temperature, then centrifuged at 4 °C at 10,000 rpm for 15 min (ROTINA 380R, Hettich, Germany). Starch was measured as glucose with the enzymatic colorimetric GOPOD method. The absorbance was read at 500 nm (PowerWave XS2, BioTek, Winooski, VT, USA). The concentration of glucose was multiplied by 0.9 to obtain the amount of starch in the samples. Total starch (TS) content was reported as mg in a 50 mg sample.

The starch fraction was calculated according to the in vitro digestibility of the starch in the samples. The percentage of starch fraction was calculated based on the study of Englyst et al. [27,28], where the amount of glucose present in the sample during the first 20 min was known as rapidly digestible starch (RDS); the difference between glucose measured at 120 min and 20 min was known as slowly digestible starch (SDS); and the amount of glucose that was not digested in 120 min was known as resistant starch (RS). The absorbance was read at 500 nm. The glucose concentration was multiplied by 0.9 to convert to the amount of starch in the samples [26].

%RDS = [(G_20_ − G_0_)/TS] × 0.9 × 100 (6)

%SDS = [(G_120_ − G_20_)/TS] × 0.9 × 100 (7)

%RS = [(TS − RDS − SDS)/TS] × 100(8)

where G_0_ denoted glucose released at time 0 min, G_20_ denoted glucose released at time 20 min, G_120_ denoted glucose released at time 120 min, and TS denoted total starch.

### 2.8. Amino-Group-Containing Residue

Digesta were collected at the end of the gastric phase and in the intestinal phase at 0, 10, 20, 30, 40, 60, 90, 120, 150, and 180 min. The amino-group residue of samples was determined by the ninhydrin assay, with slight modification [29,30]. Briefly, 20 μL of each sample was mixed with 380 μL of distilled water, followed by 200 μL of ninhydrin reagent. The mixtures were heated for 10 min in a heat block at 100 °C before cooling for 10 min. The absorbance of the mixtures was read at 568 nm using a microplate reader (PowerWave XS2, BioTek, Winooski, VT, USA). The concentration of lysine (1.56 to 200 μg/mL) was used as the standard curve.

### 2.9. Statistical Analysis

The results were expressed as means and standard error of mean (S.E.M.), *n* = 3. The obtained values were statistically analyzed using one-way analysis of variance (ANOVA) using SPSS software (Version 23, IBM, Chicago, IL, USA). The significant difference was analyzed by Duncan’s multiple range tests at *p* < 0.05.

## 3. Results and Discussion

### 3.1. Proximate Analysis

The proximate analysis of WWF and PPF is presented in Table 1. The total calorie content was 363 kcal/100 g and 374 kcal/100 g, respectively. The results demonstrated that the protein content of PPF was higher than that of WWF. Interestingly, 20% and 40% PPF chapati contributed to a marked increase in protein content compared to WWF chapati, demonstrating that PPF is a good ingredient for developing plant-based protein products. The current study also found a higher protein content in PPF than previously reported, ranging between 17.9 and 24.3 g/100 g [31,32]. This variation in protein content may be due to differences in growing conditions, methods of analysis and sampling, and storage duration and conditions [33]. As previously mentioned by Singh and colleagues [34], the total carbohydrate of PPF in this study was 60.53 g/100 g, which was less than that in WWF (71.82 g/100 g). A decreasing trend in total carbohydrate content of chapati samples was observed with an increasing PPF substitution. The total dietary fiber content of PPF was similar to that obtained in WWF. As compared to WWF chapati, PPF substituted chapati contained a greater amount of total dietary fiber. Consistently, previous studies found that PPF substitution significantly increases crude fibers in biscuits as compared to their refined wheat counterparts [17,18].

### 3.2. Color Profiles

The color attributes of flour in the chapati samples are presented in Table 2. A significant decrease in redness (a*) and an increase in yellowness (b*) were observed for PPF when compared to WWF. Furthermore, the lightness (L*) and yellowness of 40% PPF chapati were significantly higher than those of WWF chapati. The redness of the chapati was reduced only when increasing the substitution of PPF by 40%, suggesting that the color changes in chapati may be due to the effect of PPF substitution. An earlier investigation found a decreased lightness and an increased yellowness in chapati substituted with multigrain flours at 5–25% [19]. As previously mentioned, the redness of chapati significantly decreased when replacing wheat flour with white-colored finger millet flour at 25%, as compared to wheat and red-colored finger millet substituted chapati [10]. Several factors can affect the color of the product surface, such as temperature, moisture, cooking time, and the composition of reducing sugars, amino acids, or proteins on the product surface [35]. Primarily, roasting can cause browning and caramelization reactions, affecting samples’ lightness, redness, and yellowness [36]. Prior research reported that roasting peanuts within the first 5 min resulted in a slight increase in lightness and a reduction in the redness of the samples [36]. In the current study, the chapatis were also roasted on very high heat for about 2 min, which may affect the lightness and redness of PPF chapati samples in a similar manner. The calculated total color difference (∆*E*) between WWF and PPF was 1.30 ± 0.28. The total color difference from WWF chapati was 0.88 ± 0.44 and 1.92 ± 0.31 for chapati substituted with 20% and 40% PPF, respectively. As all ∆*E* values were in a range between 1 and 2, the results suggested that the color difference among samples could be detectable only by an experienced observer [22].

### 3.3. Starch Digestibility and Fraction

**Figure 1:**
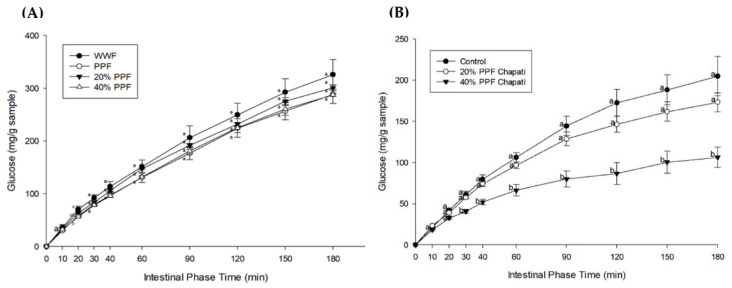
The effects of pigeon pea flour (PPF) and its partial substitution for whole wheat flour (WWF) on glucose release. The glucose profiles of (**A**) flour samples and (**B**) chapati samples. Different superscript letters for the same time interval denote statistically significant differences in the mean values among the groups at *p* < 0.05 (*n* = 3). 20% PPF: 20% pigeon pea flour substitution; 40% PPF: 40% pigeon pea flour substitution.

Figure 1A illustrates the glucose release of the flour samples during simulated digestion. A significant reduction of glucose release was observed at 20 min for PPF (56.56 ± 1.47 mg/g sample) when compared to WWF (70.35 ± 5.78 mg/g sample). Partial substitution of PPF at 20% and 40% caused a slight reduction in glucose release. Figure 1B demonstrates the glucose release of chapatis during simulated digestion. It was observed that 40% PPF chapati had significantly lower glucose release at all time points above 20 min when compared to WWF chapati. Table 3 summarizes the pGI, hydrolysis index (HI), and area under the curve (AUC) of chapati samples. The results demonstrated that the chapati with 20% and 40% PPF substitution had significantly lower pGI, %HI, and AUC values than the WWF chapati. The findings indicate that 40% of PPF chapati has low GI [33]. Similarly, a few studies reported that PPF substitution contributed to a decrease in %HI [17] and GI [18]. Prior research also found decreased levels of digested starch by replacing wheat flour with an intact cell chickpea powder at 30% and 60% in bread rolls [37]. These findings suggest that legume flours, including PPF, can be considered as wheat flour substitutes to attenuate starch digestibility in food products.

In general, the pGI value of foods depends on the parameters of SDS and RS and the degree of gelatinization [38]. Therefore, we further analyzed total starch and its fractions (RDS, SDS, and RS) as shown in Table 3. Interestingly, the starch fraction of whole wheat bread was reported at 91.7%, 4%, and 4.3% for RDS, SDS, and RS, respectively [39]. The discrepancies from the current results may be partly due to differences in sources of flour, sample preparation and storage, measurement methods, and food matrix [40]. The results also showed that the total starch content was significantly reduced in the chapati with 20% and 40% PPF substitution compared to WWF chapati. In the starch fraction, the substitution of 40% PPF did not change the RDS content, while the SDS proportion significantly decreased with the increased RS content. A recent study demonstrated that finger millet flour substitution at 25% in wheat-based chapati significantly increased the SDS and RS proportion [10]. Based on the current findings, the low pGI value of PPF chapati may result from the increase in RS proportion. Interestingly, PPF has lower amylopectin and higher amylose content when compared to WWF [41]. This finding could be explained by the difference in amylose-to-amylopectin ratio between WWF and PPF, influencing the digestibility and the physiological response of starch [42]. Therefore, the higher value of amylose in PPF can help slow down the digestion of starch into glucose, resulting in a lower %HI and GI of chapati and an increase in resistant starch proportion. Furthermore, it has been shown that dietary protein can inhibit the digestibility of starch by creating a protective layer around the starch, reducing access to carbohydrate-hydrolyzing enzymes [43,44]. López-Barón et al. reported that plant proteins caused a reduction in the in vitro starch digestibility of wheat by enhancing starch-protein interactions [45]. Therefore, the lower glucose release and the decreased SDS content with an increasing proportion of RS in PPF chapati may be partly attributed to the higher protein content in the product. Replacing or mixing flour with other ingredients such as plants is suggested to be a principal strategy of glycemic control, aiming to reduce starch digestibility, the amount of available carbohydrate for digestion, and the rate of glucose absorption [24,46,47,48]. Our findings suggest that PPF may be a promising ingredient to reduce starch digestibility and glucose release in whole wheat flat bread, which may help lower blood sugar levels. 

### 3.4. Amino-Group-Containing Residue

**Figure 2:**
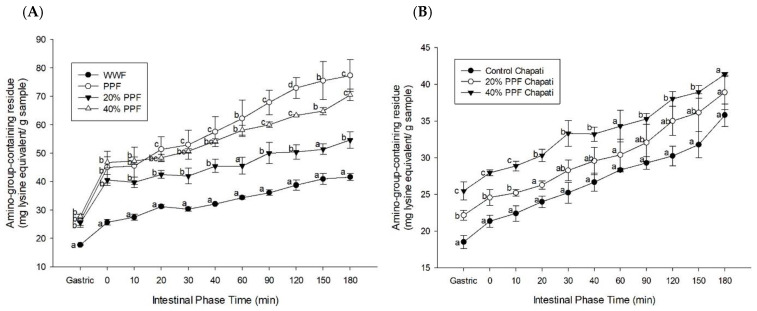
The effects of pigeon pea flour (PPF) and its partial substitution for whole wheat flour (WWF) on amino-group-containing residues equivalent to lysine: (**A**) flour samples; (**B**) chapati samples. The results are expressed as mean ± SEM, *n* = 3. ^a–d^ Different superscript letters for the same time interval denote statistically significant differences in the mean values among the groups at *p* < 0.05. 20% PPF: 20% pigeon pea flour substitution; 40% PPF: 40% pigeon pea flour substitution.

The amount of amino-group-containing compounds equivalent to lysine in the flour samples is shown in Figure 2A. In this study, the amino-group-containing residue released during simulated digestion was measured to identify whether other macronutrients and dietary fibers interfered with protein digestibility. The results showed that PPF blends had a significantly higher release of amino-group residues at most time points compared to WWF. In the gastric phase to 180 min of digestion, the amino-group residues ranged from 17.72 ± 0.18 to 41.61 ± 1.26 mg lysine/g sample for WWF and from 26.12 ± 2.26 to 77.33 ± 5.64 mg lysine/g sample for PPF. As shown in Figure 2B, chapati substituted with 20% PPF (22.15 ± 0.66 mg lysine/g sample) and 40% PPF (25.46 ± 1.23 mg lysine/g sample) had a significantly higher release of amino-group-containing compounds than that of the WWF chapati (18.51 ± 0.88 mg lysine/g sample), indicating a higher protein digestibility of PPF chapati. Similarly, prior research demonstrated that Acha-pigeon pea biscuits had a greater level of in vitro protein digestibility compared to the control white wheat biscuit [17]. The current results also showed that the increased amino-group-containing compounds were markedly released when increasing PPF substitution in flour and chapati, indicating that the substitution of PPF in chapati caused an increase in protein content and a decrease in total starch. This effect possibly impairs the interference of starch with protein digestion, which contributes to more accessibility of protein-hydrolyzing enzymes and increases the release of amino-group-containing residues in PPF samples. In contrast, a previous report found that protein could interact with starch and form a protein–starch matrix, which decreased access of enzymes to protein [49]. According to the current findings, protein in PPF is rapidly digested within 2 h of simulated digestion without the effect of total starch content, which may help enhance amino acid absorption and protein synthetic machinery in the body. A fast-digestible protein, such as whey protein, is also digested within 1–2 h, which may improve protein synthesis and oxidation [50].

The food industry and current research exhibit an increasing trend in using plant proteins derived from grains, seeds, and legumes as substitutes for animal proteins [51]. The plant protein source must have good functional properties, high nutritional value, and protein digestibility that support long-term public health goals. Wheat typically has an adequate amount of sulfur-containing amino acids but is low in lysine [52], whereas pigeon pea is high in lysine and limited in sulfur-containing amino acids [53]. Combining different vegetal sources of protein with complementary indispensable amino acids practically contributes to a better quality of protein [54]. The current findings suggest that both PPF chapati formulations can be useful to target the gap of high protein digestibility. Although using PPF in food products can fulfill the majority of essential amino acid requirements, the protein quality of products may still be inferior to that from animal sources [42]. Therefore, seeking a suitable plant protein combination has brought further challenges in improving the protein quality of PPF-substituted products to be comparable to animal-based proteins.

## 4. Conclusions

The development of chapati with whole wheat–pigeon pea flour blends could improve nutritional characteristics, while its color profile was relatively well maintained. Examining starch and protein digestibility together provides an overall understanding of how nutrient changes from pigeon pea flour substitution affect chapati digestion. A decrease in starch digestibility in chapati substituted pigeon pea flour was found, corresponding to an increment in resistant starch proportion. Digestible protein release from chapati was increased with the pigeon pea flour substitution, which could enhance protein absorption. These findings can be used as a starting point for further research on the usage of pigeon pea flour in wheat products. This calls for future investigation into the mechanistic formation of resistant starch, protein bioaccessibility, amino acid profile, and release during digestion to give a deeper insight. From the foregoing, pigeon pea can be considered as one of the plant-based protein alternatives that can be applied to foods to reduce starch digestibility and improve protein content.

## Figures and Tables

**Table 1:** Proximate composition (dry matter basis) and moisture (as sampled basis) of pigeon pea flour (PPF), whole wheat flour (WWF) and the chapatis per 100 g sample.

Experiments	WWF	PPF	WWF Chapati	20% PPF Chapati	40% PPF Chapati
Total calories (kcal)	363.32 ± 0.33 ^b^	374.06 ± 0.98 ^a^	274.08 ± 0.48 ^c^	266.25 ± 0.19 ^d^	265.41 ± 1.00 ^d^
Total carbohydrate (g)	71.82 ± 0.02 ^a^	60.53 ± 1.55 ^b^	55.73 ± 0.3 ^c^	52.15 ± 0.40 ^d^	50.24 ± 0.03 ^e^
Ash (g)	1.24 ± 0.03 ^b^	1.51 ± 0.10 ^a^	0.97 ± 0.02 ^e^	1.14 ± 0.01 ^d^	1.35 ± 0.01 ^c^
Total fat (g)	2.44 ± 0.17 ^b^	3.06 ± 0.06 ^a^	1.60 ± 0.04 ^c^	1.58 ± 0.03 ^c^	1.69 ± 0.02 ^c^
Protein (N × 6.25, g)	13.52 ± 0.28 ^b^	26.10 ± 1.16 ^a^	9.19 ± 0.52 ^d^	10.88 ± 0.37 ^c^	12.31 ± 0.32 ^c^
Total dietary fiber (g)	10.08 ± 0.19 ^a^	10.41 ± 0.02 ^a^	10.34 ± 0.17 ^a^	11.02 ± 0.04 ^b^	11.08 ± 0.11 ^b^
Moisture (g)	10.98 ± 0.15 ^a^	8.80 ± 0.23 ^b^	32.51 ± 0.16 ^d^	34.26 ± 0.01 ^c^	34.41 ± 0.26 ^c^

The results are expressed as mean ± SEM, *n* = 3. ^a–e^ Different superscript letters in the same row denote statistically significant differences in the mean values at *p* < 0.05. WWF: 100% whole wheat flour; PPF: pigeon pea flour; 20% PPF: 20% pigeon pea flour substitution; 40% PPF: 40% pigeon pea flour substitution.

**Table 2:** Color profiles of pigeon pea flour (PPF), whole wheat flour (WWF), and the chapatis.

Samples	*L* *	*a* *	*b* *
WWF	36.96 ± 0.32 ^a^	1.31 ± 0.02 ^c^	6.26 ± 0.08 ^d^
PPF	35.98 ± 0.35 ^a^	0.78 ± 0.02 ^d^	6.84 ± 0.12 ^c^
WWF Chapati	21.79 ± 0.65 ^c^	3.10 ± 0.09 ^a^	8.63 ± 0.06 ^b^
20% PPF Chapati	22.55 ± 0.47 ^c^	2.77 ± 0.05 ^ab^	8.77 ± 0.10 ^ab^
40% PPF Chapati	23.49 ± 0.32 ^b^	2.47 ± 0.14 ^b^	9.17 ± 0.15 ^a^

The results are expressed as mean ± SEM, *n* = 3. ^a–d^ Different superscript letters in the same column denote statistically significant differences in the mean values at *p* < 0.05. WWF: 100% whole wheat flour; PPF: pigeon pea flour; 20% PPF: 20% pigeon pea flour substitution; 40% PPF: 40% pigeon pea flour substitution.

**Table 3:** Predicted glycemic index (pGI), hydrolysis index (HI), glucose area under the curve (AUC), and starch fractions of chapati samples.

Samples	pGI	%HI	AUC (mg·min/g Sample)	TS (g/50 g Sample)	%RDS	%SDS	%RS
WWF Chapati	51.55 ± 0.20 ^a^	21.57 ± 0.58 ^a^	30,644.34 ± 818.86 ^a^	21.55 ± 0.27 ^a^	17.87 ± 1.30 ^a^	47.64 ± 2.32 ^a^	34.49 ± 3.25 ^a^
20% PPF Chapati	49.55 ± 0.68 ^b^	17.93 ± 1.24 ^b^	25,475.88 ± 1767.09 ^b^	20.54 ± 0.04 ^b^	15.38 ± 1.07 ^a^	40.59 ± 3.37 ^a^	44.02 ± 4.43 ^a^
40% PPF Chapati	47.19 ± 0.31 ^c^	13.62 ± 0.56 ^c^	19,356.81 ± 795.26 ^c^	18.55 ± 0.09 ^c^	14.54 ± 0.98 ^a^	27.67 ± 3.32 ^b^	57.99 ± 2.67 ^b^

The results are expressed as mean ± SEM, *n* = 3. ^a–c^ Different superscript letters in the same column denote statistically significant differences in the mean values at *p* < 0.05. WWF: 100% whole wheat flour; PPF: pigeon pea flour; 20% PPF: 20% pigeon pea flour substitution; 40% PPF: 40% pigeon pea flour substitution. TS: total starch; RDS: rapidly digestible starch; SDS: slowly digestible starch; RS: resistant starch.

## Data Availability

Data are contained within the article.
